# Emergency Revascularization for Acute Vertebral Artery Occlusion Accompanied by Systemic Lupus Erythematosus: A Case Report

**DOI:** 10.7759/cureus.60733

**Published:** 2024-05-21

**Authors:** Junpei Nagasawa, Makiko Ogawa, Hiromi Konaka, Masaru Yanagihashi, Osamu Kano

**Affiliations:** 1 Neurology, Toho University Faculty of Medicine, Tokyo, JPN

**Keywords:** cerebral infarction, vasculitis, stroke, systemic lupus erythematous (sle), mechanical thrombectomy (mt)

## Abstract

The efficacy of mechanical thrombectomy (MT) for acute ischemic stroke has been established, but there are few reports on the effectiveness of MT for stroke patients with collagen disease. We report the case of a systemic lupus erythematosus (SLE) patient with cerebral infarction who underwent MT. A 48-year-old woman had been diagnosed with SLE for 30 years. She visited our hospital because of dizziness from the day before, but when she arrived at the hospital parking lot, she developed vomiting and impaired consciousness. An MRI revealed increased cerebellar hemisphere infarction and magnetic resonance angiography (MRA) did not visualize the right vertebral artery or basilar artery. Urgent cerebral angiography was performed, and angiography of the right vertebral artery revealed occlusion of the V4 segment of the vertebral artery. In addition to these angiographic findings, the patient also had impaired consciousness and was judged to be in need of emergency revascularization treatment. We performed an MT using a stent retriever. Immediately after the angiography examination, reperfusion to the basilar artery and severe stenosis of the right vertebral artery were noted. Therefore, percutaneous transluminal angioplasty (PTA) and stent placement for vertebral artery stenosis were done. This procedure successfully maintained the patency of the vertebral artery and blood flow to the basilar artery. Her consciousness improved; she only had mild nausea and no remarkable neurological findings.

## Introduction

Systemic lupus erythematosus (SLE) is an autoimmune disease characterized by systemic inflammatory lesions caused by the deposition of immune complexes, such as anti-DNA antibodies, in tissues. It is an important cause of stroke, whose prevalence among patients with SLE is 3.1% and incidence is 1.25 per 1000 annually; more than half of these cases are acute ischemic stroke (AIS)[1].

Systemic lupus erythematosus can cause ischemic strokes through multiple pathophysiological mechanisms. Cerebrovascular events account for 10% to 15% of SLE deaths [2]. Therefore, treating stroke in patients with SLE is very important. Evidence of the effectiveness of mechanical thrombectomy (MT) for cerebral infarction with large-vessel occlusion (LVO) has been established in recent years. However, there are few reports of patients with SLE, and the effectiveness of MT in patients with SLE is unknown. We encountered a patient with SLE who developed cerebral infarction due to acute vertebral artery occlusion and underwent emergency mechanical revascularization.

## Case presentation

A 48-year-old woman had been diagnosed with SLE 30 years ago and treated with prednisolone (PSL) 6 mg, azathioprine 100 mg, and cyclosporine 200 mg. All medicines were taken orally. The SLE activity was relatively stable, with an SLE disease activity index score of 8 points. She had been taking PSL 20 mg orally during a period of high SLE activity 10 years ago but had been taking less than 8 mg PSL for the past few years. She had no common risk factors for arteriosclerosis, such as high blood pressure, diabetes, and dyslipidemia, other than a history of smoking 10 cigarettes a day. 

**Figure 1:**
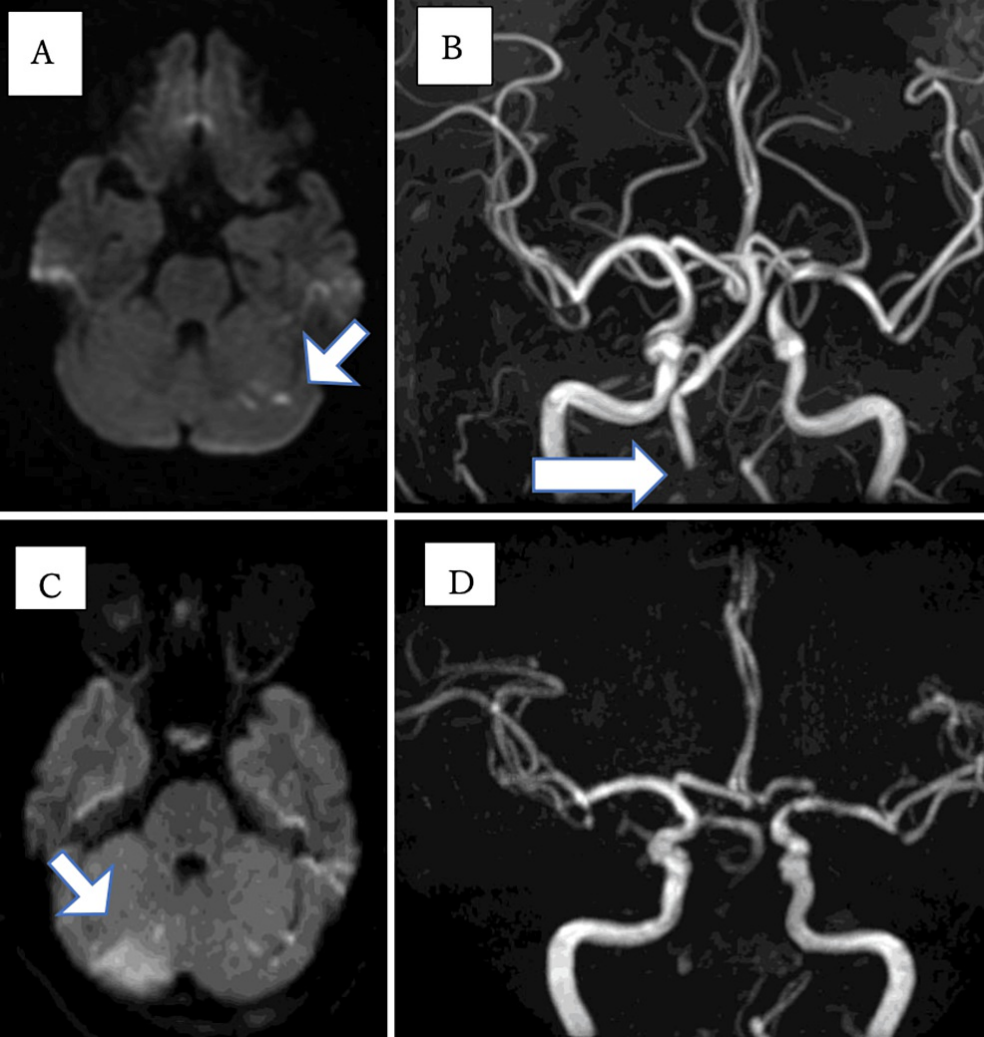
The MRI taken at onset (A, B) and the MRI taken at our hospital (C, D) A: Acute cerebral infarction in the left cerebellar hemisphere (arrow) and bilateral occipital lobes; B: MRA revealed partial stenosis of the right vertebral artery (arrow); C: MRI performed the next day shows increased cerebellar hemisphere infarction (arrow); D: MRA does not visualize the right vertebral artery and basilar artery MRA: Magnetic resonance angiography

**Figure 2:**
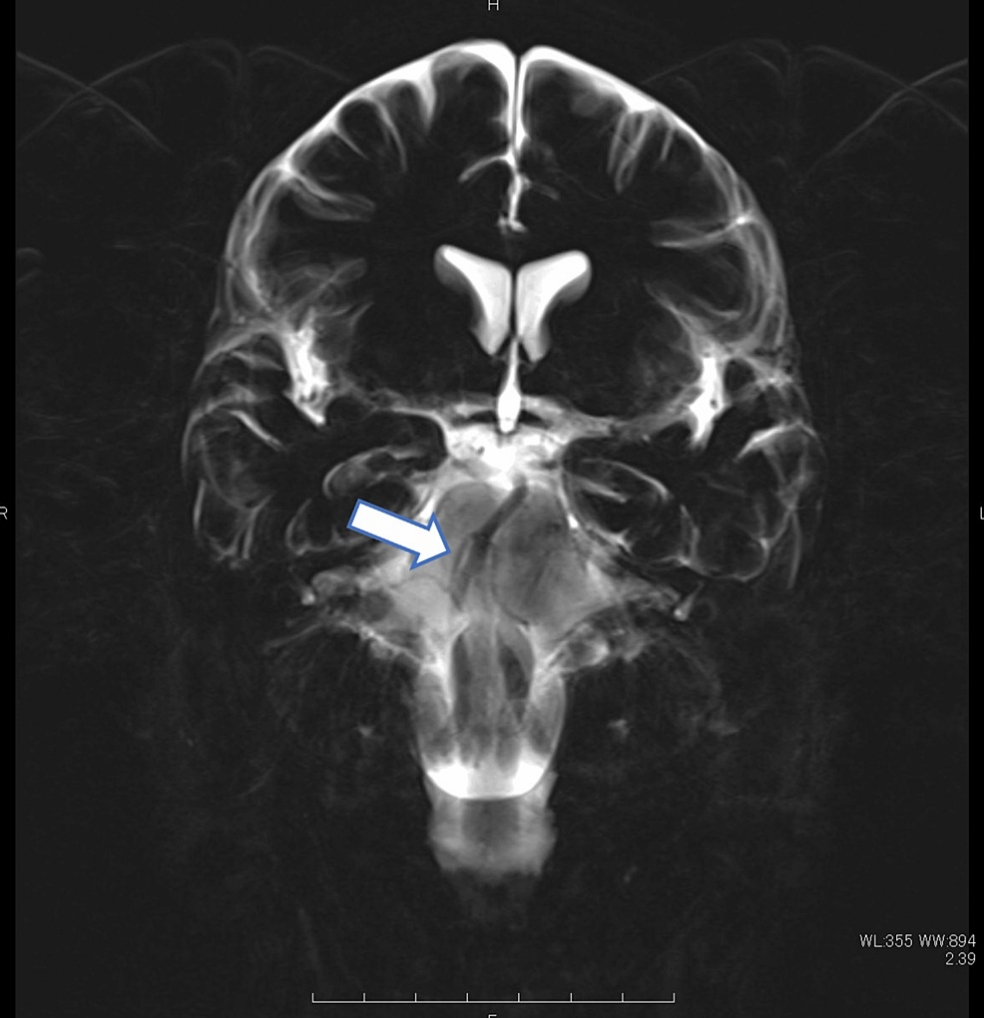
The BPAS MRI The BPAS MRI shows no dilated findings of the right vertebral artery (arrow). BPAS: Basi-parallel anatomical scanning

One day when she woke up, she had dizziness. On the same day, she visited a neurosurgery clinic and underwent MRI. The MRI revealed acute cerebral infarction in the left cerebellar hemisphere and bilateral occipital lobes (Figure 1A), and magnetic resonance angiography (MRA) revealed partial stenosis of the right vertebral artery (Figure 1B). The next day, when she arrived at our hospital parking lot, she developed vomiting and impaired consciousness. At the time of onset, she was in a coma, but after about 15 minutes, her consciousness improved slightly. Physical findings on arrival were as follows: blood pressure 144/86 mmHg; heart rate 80 beats/min; respiratory rate 16 times/min; saturation of peripheral oxygen (SpO2) 99%; Glasgow coma scale (GCS) grade E3V4M6. While the MRI revealed increased cerebellar hemisphere infarction (Figure 1C), the MRA did not visualize the right vertebral artery and basilar artery (Figure 1D). The basi-parallel anatomical scanning (BPAS) MRI revealed no abnormal findings in the right vertebral artery (Figure 2).

**Figure 3:**
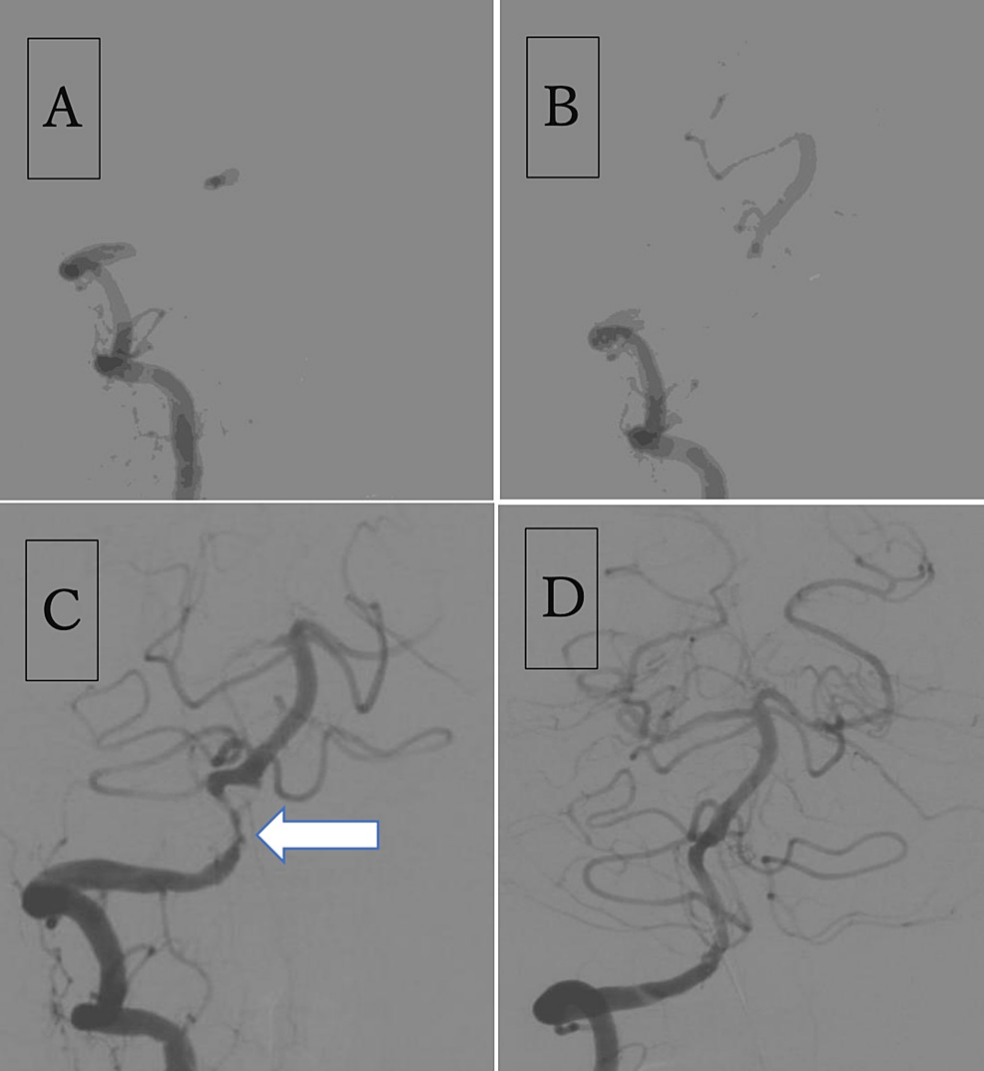
Emergency revascularization for acute right vertebral artery occlusion A: Angiography of the right vertebral artery showing occlusion of the V4 segment of the vertebral artery; B: Angiography from the basilar artery after the lesion crossing the vertebral artery occlusion revealed no occlusion of the basilar artery; C: Angiography showing reperfusion to the basilar artery and right vertebral artery stenosis (arrow); D: The final angiogram reveals no stenosis of the vertebral artery and good blood flow to the basilar artery.

An urgent cerebral angiography was performed to accurately evaluate the cerebral blood flow. Angiography of the right vertebral artery revealed occlusion of the V4 segment of the vertebral artery (Figure 3 A). Angiography of the left vertebral artery revealed a posterior inferior cerebellar artery end pattern in the left vertebral artery. Collateral flow from the internal cervical artery to the posterior circulation via the posterior communicating artery could not be confirmed on either side. Therefore, we concluded that there was no effective blood flow to the posterior circulation owing to right vertebral artery occlusion. In addition to these angiographic findings, the patient also had impaired consciousness and was judged to be indicated for emergency revascularization treatment. 

A Trevo NXT 4 mm × 28 mm (Stryker Corp., Kalamazoo, MI, USA) was deployed at the obstructed part of the right vertebral artery through a Trevo Track 21 microcatheter (Stryker Corp.). Immediately after the angiography examination, reperfusion to the basilar artery and severe stenosis of the right vertebral artery were noted (Figure 3 C). Later, the right vertebral artery stenosis caused poor blood flow to the basilar artery and was occluded again. Therefore, percutaneous transluminal angioplasty (PTA) for vertebral artery stenosis was considered necessary. The PTA was performed with a Gateway OTW, 2.0×12 mm, microballoon catheter (Stryker Corp.). The balloon catheter was inflated to a nominal pressure (6.0 atm) for 60 s. This procedure resulted in temporary dilation of the artery stenosis, but restenosis appeared 5 minutes later. Next, the balloon catheter was inflated to the rated burst pressure (10.0 atm) for 60 s. However, the dilation of the stenosis was temporary, and restenosis reappeared 5 minutes after angiography. Therefore, stenting was required to maintain blood flow to the basilar artery. A Neuroform Atlas 3×12 mm (Stryker Corp.) was deployed through a Trevo Track 21 microcatheter, and post-dilation was performed using a SHIDEN 2.5×20 mm microballoon catheter (Kaneka Corp., Tokyo, Japan). Subsequently, angiography was performed every 5 minutes for 30 minutes; this procedure successfully maintained the patency of the vertebral artery and blood flow to the basilar artery (Figure 3 D). The following day, the patient's consciousness improved. She only had mild nausea and there were no remarkable neurological findings.

The patient's laboratory test results are displayed in Table 1. Cerebrospinal fluid (CSF) analysis showed 3 WBCs/μl, protein 55 mg/dL, and normal glucose 52 mg/dL. The thrombus retrieved by MT was a red thrombus, and pathological examination revealed that it was a thrombus containing a mixture of fibrin and red blood cells. Blood tests did not reveal the presence of antiphospholipid antibodies; thus, the patient did not meet the diagnostic criteria for antiphospholipid syndrome (APS). Transthoracic echocardiography revealed no endocarditis. Cerebrospinal fluid examination revealed no increase in the cell counts or protein levels. Follow-up angiography, CT angiography, and MRA showed no restenosis of the right vertebral artery. The patient was discharged after 15 days of hospitalization. At discharge, she had a modified Rankin scale score of 0.

**Table 1:** Laboratory test results CRP: C-reactive protein, LDL: Low-density lipoprotein, HDL: High-density lipoprotein, HbA1c: Glycated hemoglobin, PT: Prothrombin time, INR: International normalized ratio, aPTT: Activated partial thromboplastin time, C3: Complement component 3, C4: Complement component 4, SS-A: Sjögren's syndrome type A, SS-B: Sjögren's syndrome type B

Investigations	Value
White blood cells	9.0 × 103/μl
Neutrophils	5.3 × 103/μl
CRP	0.5 mg/dl
Triglyceride	98 mg/dl
LDL-cholesterol	117 mg/dl
HDL-cholesterol	52 mg/dl
HbA1c	6.0%
Glucose	84 mg/dl
PT INR	1.1 (<1.2)
aPTT	26.5 sec
D-dimer	0.5 μg/ml
Anti-cardiolipin antibodies IgG	<0.7 U/m
Anti-cardiolipin β2-glycoprotein complex antibody	≤8 U/ml
Lupus anticoagulant (diluted Russell’s Viper venom time)	0.9 sec
Anti-dsDNA antibody	38 IU/ml
C3	85 mg/dl
C4	7 mg/dl
SS-A and SS-B antibodies	Negative

## Discussion

Systemic lupus erythematosus increases the risk of ischemic stroke, especially in people aged <50 years [3,4]. Cerebral infarction is more likely to occur in the first year or 10 years after diagnosis of SLE [2]. Furthermore, individuals with SLE have a lower rate of survival after stroke than the general population [5]. Only two cases of MT for cerebral infarction in patients with SLE have been reported, and there have been no reports of PTA and stenting, as in this case (Table 2) [1,6]. 

**Table 2:** Past reports of patients with SLE who underwent emergency mechanical revascularization due to large-vessel occlusion BA: Basilar artery, MCA: Middle cerebral artery, MT: Mechanical thrombectomy, mRS: Modified Rankin scale, SLE: Systemic lupus erythematosus, APS: Antiphospholipid syndrome

Authors	Year	Age/sex	Lesion	Etiology	APS	Treatment	mRS score
Jiaxia et al.^[6^^]^	2020	55/female	BA	Embolic	Positive	MT	mRS 2
Han et al. ^[^^1]^	2021	38/female	Left MCA	Artery stenosis	Negative	MT	mRS 1

The mechanisms underlying cerebral infarction in patients with SLE are assumed to be endocarditis, autoimmune-mediated vasculitis, inflammation-mediated atherosclerosis, or blood hypercoagulability secondary to antiphospholipid antibody (aPL) emergence [7,8]. In pathological examination, cases associated with vasculitis are relatively few, and thrombus, vascular wall degeneration, and endothelial proliferation are often observed [9]. In this case, cerebral infarction might have developed due to autoimmune-mediated vasculitis or inflammation-mediated atherosclerosis, because angiography revealed stenosis of the artery itself, and there were no findings suggestive of endocarditis or APS on transthoracic echocardiography or blood sampling. Arterial dissection is also considered for juvenile vertebral artery occlusion.

However, in this case, no findings suggestive of dissection (such as the pearl and string sign, double lumen, intramural hematoma, and an intimal flap) were found on angiographic or MRI and BPAS images, and vertebral artery dissection was ruled out. Unlike embolic infarction, in infarctions associated with arterial stenosis, even if a temporary recanalization can be obtained with MT alone, artery stenosis is often re-occluded in a short time. Therefore, the effective way to treat such cases is not only to remove the thrombus but also to perform PTA and stenting for stenosis to maintain the patency of the blood vessel.

As already mentioned, there have been no reports of PTA and stenting in SLE patients. However, there are reports of PTA and stenting for intracranial arterial stenosis due to giant cell arteritis. The results of this systematic review show that immediate angiographic improvement in luminal diameter was observed in all cases. Regarding complications, procedure-related arterial dissection was observed in 10.5%, and none were associated with new neurologic deficits [10]. Both PTA and stenting may be effective for vascular stenosis related to collagen disease or vasculitis, but it is necessary to collect more cases related to SLE in the future.

## Conclusions

In patients with SLE, severe cerebral infarction associated with large-vessel occlusion can occur. It can occur not only due to thromboembolism but also due to vasculitis. Therefore, it is important to consider the mechanism underlying the infarction and not miss the indication for emergency treatment, including MT and PTA.
